# Drug Screening Implicates Chondroitin Sulfate as a Potential Longevity Pill

**DOI:** 10.3389/fragi.2021.741843

**Published:** 2021-09-08

**Authors:** Collin Y. Ewald

**Affiliations:** Laboratory of Extracellular Matrix Regeneration, Department of Health Sciences and Technology, Institute of Translational Medicine, ETH Zürich, Switzerland

**Keywords:** chondroitin sulfate, supplement, healthy aging, longevity, anti inflammatory, extracellar matrix, drug discovery, matreotype

## Abstract

Discovering compounds that promote health during aging (“geroprotectors”) is key to the retardation of age-related pathologies and the prevention of chronic age-related diseases. In *in-silico* and model organisms’ lifespan screens, chondroitin sulfate has emerged as a geroprotective compound. Chondroitin sulfate is a glycosaminoglycan attached to extracellular matrix proteins and is naturally produced by our body. Oral supplementation of chondroitin sulfate shows a high tolerance in humans, preferable pharmacokinetics, a positive correlation with healthy human longevity, and efficacy in deceleration of age-related diseases in randomized clinical trials. We have recently shown that chondroitin sulfate supplementation increases the lifespan of *C. elegans*. Thus, chondroitin sulfate holds the potential to become a geroprotective strategy to promote health during human aging. This review discusses the two major potential mechanisms of action, extracellular matrix homeostasis and inhibition of inflammation, that counteract age-related pathologies upon chondroitin sulfate supplementation.

## Introduction

### Predicted Longevity Drug-Protein Targets Reveal Chondroitin

**FIGURE 1:**
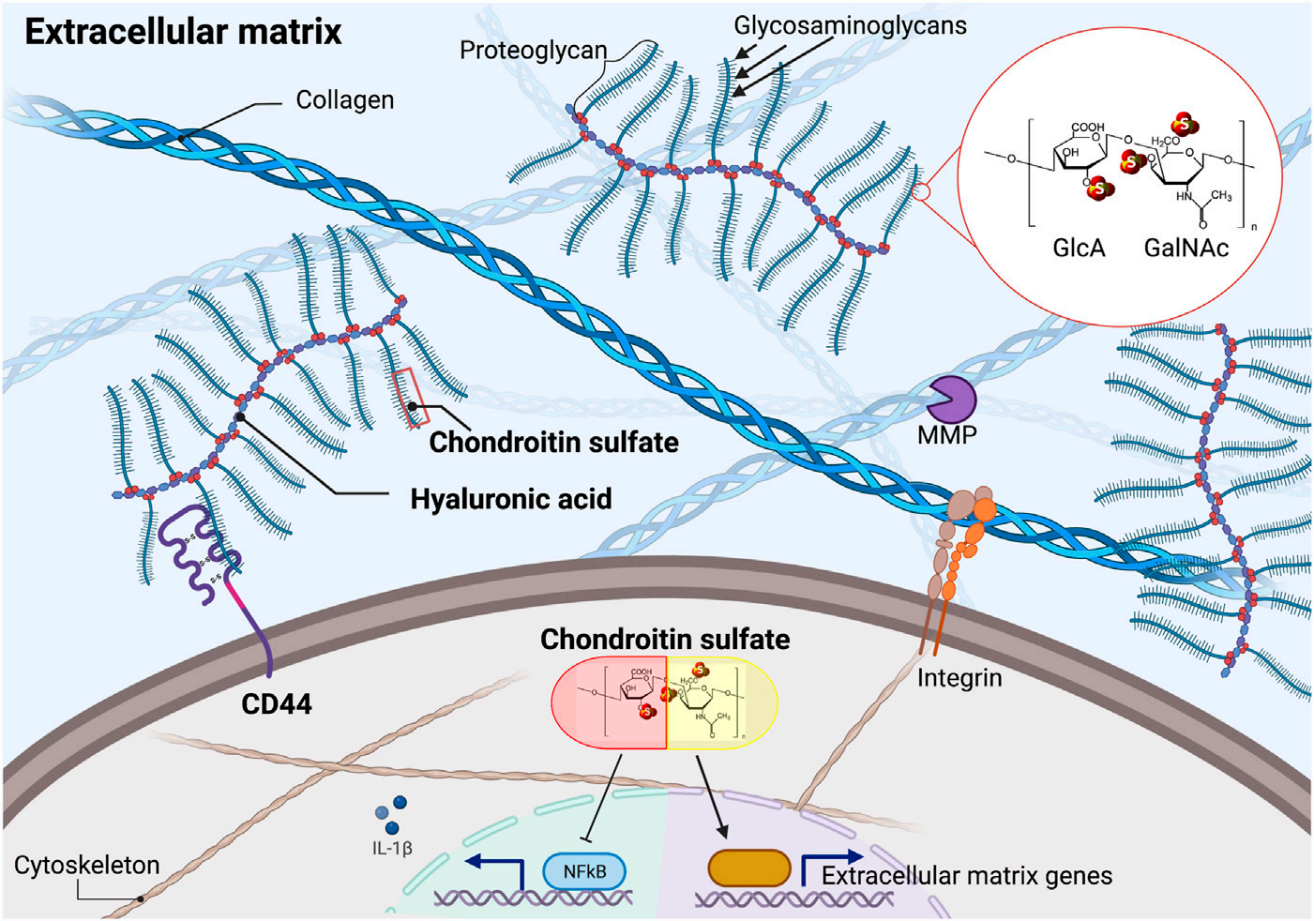
Chondroitin sulfate in the extracellular matrix and cellular effects of supplementation. Chondroitin sulfate consists of repeating units of D-glucuronic acid (GlcA) and N-acetyl galactosamine (GalNAc). Naturally occurring chondroitin sulfate only shows one of the three sulfates at the indicated (S) positions, resulting in distinct isomers. Chondroitin sulfate polymers are the building blocks of proteoglycans that can be attached to the hyaluronic acid polymers. Supplementation of chondroitin sulfate blocks NF-ƙB mediated inflammation and stimulates extracellular matrix homeostasis. MMP = matrix metalloproteinase. Illustration not drawn to scale.

How to identify compounds that retard age-related pathologies and promote health during aging? There are many approaches to identify geroprotective compounds, spanning from *in-silico* and cell-based drug screening to direct lifespan assays in model organisms (Barardo et al., 2017; Bakula et al., 2018; Janssens et al., 2019; Kusumoto et al., 2021; Statzer et al., 2021). One of the largest screens assessed 1,300 compounds on about 20,000 mice performing full lifespans and yielded five longevity-promoting compounds (WO2018075641A1 and US 20200254006 A1). Using *C. elegans,* more than 100,000 compounds have been screened collectively across multiple studies, and about 100 compounds have been identified that increase *C. elegans* lifespan (Petrascheck et al., 2007; Lucanic et al., 2013; Ye et al., 2014; Kim and Lee, 2019; Statzer et al., 2021). Pathway analysis of these discovered longevity compounds showed enrichment for TGFβ pathway, chondroitin, and heparan sulfate biogenesis as potential drug-protein targets (Liu et al., 2016). In our drug screens, we also identified chondroitin sulfate (Statzer et al., 2021). Chondroitin sulfate is a naturally occurring sulfated glycosaminoglycan usually attached to extracellular matrix proteins (Figure 1). Thereby, it is abundantly found in connective tissues, such as cartilage that cushions the ends of the bones within the joints, skin, blood vessels, ligaments, and tendons, but also in other organs, such as the brain (Jerosch, 2011; Kwok et al., 2012). Chondroitin sulfate is a popular and widely used supplement that is well-tolerated, with no adverse effects above placebo, and is likely very safe, allowing long-term treatment (Hathcock and Shao, 2007). Thus, making chondroitin sulfate an ideal candidate to develop further into a geroprotective compound.

### *In-vivo* Drug Screen *C. Elegans* Identifies Glycosaminoglycans to Extend Lifespan

While examining previously established longevity compounds, such as metformin and rapamycin, we noticed a robust extracellular matrix transcriptional signature as a cellular response to the drug (Statzer et al., 2021). Moreover, in 41 out of 47 known longevity compounds (*i.e.,* almost 90%) we examined, extracellular matrix gene expression was significantly altered (Statzer et al., 2021), suggesting remodeling of the extracellular matrix elicited by these drugs (Ewald, 2020). Therefore, in contrast to previous drug screens, we centered our *in-silico* analysis around the extracellular matrix (Ewald, 2020). We analyzed the human Genotype-Tissue Expression (GTEx) transcriptomic data combined with eight different expression profile datasets to define the aging matreotype across tissues (Statzer et al., 2021). The aging matreotype is a list of 99 extracellular matrix genes that either decline or increase in expression during human aging (Statzer et al., 2021). Then we probed the cMap expression profiles of about 1,300 drugs for a “youthful” matreotype signature and identified 185 compounds, of which 24 have previously been shown to increase lifespan in model organisms (Statzer et al., 2021).

A challenge in validating compounds for healthy aging is to identify the beneficial drug dose. We developed a novel *in-vivo* surrogate marker for longevity, using collagen homeostasis as a read-out in *C. elegans* (Statzer et al., 2021). With this reporter, we examined the unexplored area of proteoglycans and glycosaminoglycans as they were identified as major drug targets across many geroprotective drugs (Liu et al., 2016). These glycosaminoglycans, such as hyaluronic acid, chondroitin sulfate, and glucosamine, are major components of extracellular matrix proteins, found in cartilage and synovial fluid, and are naturally produced by the body or can be supplemented by diet (Jerosch, 2011). We found that supplementing hyaluronic acid and chondroitin sulfate increased *C. elegans* lifespan by 25–35% and 23–28%, respectively (Statzer et al., 2021). Previous work by the Ristow lab had shown that supplementation with glucosamine increased mouse lifespan and also *C. elegans* lifespan by 8–12% (Weimer et al., 2014). Thus, oral uptake of these glycosaminoglycans promotes healthy aging and longevity in model organisms.

### Biosynthesis of Chondroitin and Relationship to Glucosamine and Hyaluronic Acid

Glucosamine is a precursor and the rate-limiting step in the synthesis of chondroitin polymers, which are the building blocks of the side chains of several proteoglycans (Figure 1) (McCarty et al., 2000; Mikami and Kitagawa, 2013). In particular, chondroitin consists of long polysaccharides of 20–200 repeating units of N-acetyl galactosamine (GalNAc) and d-glucuronic acid (GlcA), which can be sulfated at three different positions resulting in distinct isomers (Figure 1) (Mikami and Kitagawa, 2013). Chondroitin sulfate is usually of 10–50 kDa molecular weight and is extracted from cartilaginous tissues from pigs, cows, birds, and sharks (Jerosch, 2011). Glucosamine and chondroitin sulfate may facilitate hyaluronic acid production (McCarty et al., 2000).

Following oral uptake, glucosamine, chondroitin sulfate, and hyaluronic acid get transported to the target tissue in animal studies; molecules are safe and show high-quality evidence for their effectiveness in randomized clinical trials (Balogh et al., 2008; Bruyère et al., 2008; Jerosch, 2011; Garantziotis and Savani, 2019). However, hyaluronic acid is a huge polymer that is poorly uptaken by the body and showed variable effects in randomized clinical trials (Kalman et al., 2008). Furthermore, hyaluronic acid needs to be broken down by hyaluronidase TMEM2 to protect against protein misfolded endoplasmic reticulum stress in human fibroblasts and promote *C. elegans* longevity (Schinzel et al., 2019). Glucosamine is a smaller monomer but impairs glucose metabolism and increases lifespan in part through glucose restriction and mitochondrial reactive oxygen species-induced hormesis (Weimer et al., 2014). On the other hand, chondroitin sulfate is a slow-acting drug (Bruyère et al., 2008) that has supportive evidence to modify cartilage structure in randomized clinical trials and is repeatedly recommended for the last 20 years by the European League Against Rheumatism (EULAR) (Jordan et al., 2003; Kloppenburg et al., 2018). Furthermore, the combination of glucosamine with chondroitin sulfate showed synergistic and additive effects for osteoarthritis *in vitro, in vivo*, and in randomized clinical trials (Clegg et al., 2006; Jerosch, 2011), suggesting distinct and overlapping modes-of-action. Hence, I focus here on the potential underlying mode-of-actions and molecular mechanisms promoted by supplementation with chondroitin sulfate.

## Potential Mechanisms Promoted by Chondroitin Sulfate

### Mechanism 1: Evidence of Chondroitin Sulfate Stimulating Extracellular Matrix Protein Homeostasis

During aging, the balance of extracellular matrix biosynthesis and degradation becomes dysregulated (Ewald, 2020). Genetic alterations of extracellular matrix genes cause diverse phenotypes and diseases (Huxley-Jones et al., 2008; Statzer and Ewald, 2020). While many chronic age-dependent diseases show increased inflammation and fibrotic collagen deposition (Wick et al., 2013; Bonnans et al., 2014; Teuscher et al., 2019; Haefke and Ewald, 2020; Karsdal et al., 2020), a general signature of extracellular matrix aging is the progressive decline in collagen biosynthesis and an increase of extracellular protease activity across species (Ewald, 2020). For instance, in human skin, collagen, elastic fibers, laminin, and integrin levels progressively decline during aging (Shuster et al., 1975; Bosset et al., 2003; Farage et al., 2008; Sugawara et al., 2008; Giangreco et al., 2009; Kanaki et al., 2016). The general decline of collagen biosynthesis might be driven by the senescence of fibroblasts and the loss of stem cell maintenance (Varani et al., 2006). Counteracting this age-dependent decline of collagen synthesis by prolonging collagen expression is sufficient to increase the lifespan of *C. elegans* (Ewald et al., 2015), suggesting that extracellular protein homeostasis is a novel yet unexplored mechanism to promote health during aging (Ewald, 2020).

Since domains of extracellular matrix proteins and remodeling enzymes are well conserved across species, particularly the active domains where drug target sites are preferentially located, the dynamic process of extracellular matrix remodeling has emerged as an attractive drug target (Huxley-Jones et al., 2008). *In-silico* analysis predicting chondroitin sulfate drug targets revealed mostly components for biosynthesis and degradation of chondroitins, such as chondroitinase (GALNS), sulfotransferase (CHST11), chondroitin/hyaluronic acid receptor (CD44), hyaluronidase (HYAL1, 2), and enzymes that remodel the extracellular matrix, such as matrix metalloproteinases (MMP1, 3, 16, 24) (Lila et al., 2018). In mice, deletion of chondroitin 6-sulfotransferase (CHST3) results in an abnormal extracellular matrix in the brain, accelerated brain aging, and memory impairments (Yang et al., 2021). Overexpression of CHST3 improved memory in old mice, suggesting the importance of maintaining proper chondroitin sulfate levels to promote neuroplasticity during aging (Yang et al., 2021). Furthermore, endogenous chondroitin sulfate is essential for maintaining embryonic stem cell pluripotency *via* binding to E-cadherin cell adhesion and RhoA and ERK1/2 downstream signaling (Izumikawa et al., 2014). Hence, endogenous chondroitin sulfate metabolism is linked to extracellular protein homeostasis.

In rats, endogenous chondroitin sulfate levels decline with age, probably due to the loss of chondrocytes (Honda et al., 1979). Interestingly, supplementing chondroitin sulfate increased chondrocyte cell proliferation in a dose-dependent manner (Jerosch, 2011; López-Senra et al., 2020). Moreover, supplementing *C. elegans* with chondroitin sulfate retarded the progressive decline of collagen renewal and increased lifespan (Statzer et al., 2021). In proteomics analysis of osteoarthritis patient-derived chondrocytes, *ex-vivo* chondroitin sulfate supplementation mainly resulted in remodeling of extracellular proteins and some inflammatory-associated proteins (Calamia et al., 2012a). This is consistent with many animal and human studies showing that chondroitin sulfate supplementation inhibits cartilage destruction and stimulates proteoglycan production for bolstering the connective tissues (Bassleer et al., 1998; Omata et al., 2000; Tahiri et al., 2007; Huskisson, 2008; Taniguchi et al., 2011; Martel-Pelletier et al., 2015; Sukhikh et al., 2020). Taken together, supplementing with chondroitin sulfate tips the balance towards prolonged extracellular matrix protein homeostasis, a requisite for healthy aging (Figure 1).

### Mechanism 2: Evidence of Chondroitin Sulfate in Suppressing Inflammation

Endogenous chondroitin sulfate is essential to keep inflammation in check. A spontaneously-arisen deleterious mutation in the chondroitin sulfate synthase 1 (Chsy1) resulted in lower chondroitin sulfate levels leading to chronic inflammation and shortening of mouse lifespan (Macke et al., 2020). On the other hand, chondroitin sulfate supplementation reduces chronic inflammation. For instance, in a randomized, double-blind, placebo-controlled, clinical trial on 18 placebo and 18 glucosamine and chondroitin supplemented healthy adults, chondroitin with glucosamine significantly lowered serum inflammation biomarker C-reactive Protein and substantially remodeled the extracellular matrix detected by the blood plasma proteomic arrays (Navarro et al., 2015). Similarly, chondroitin sulfate intake was associated with a reduction in C-reactive Protein concentration in the blood (Kantor et al., 2020), suggesting a decrease in inflammation.

Induction of an inflammatory response by stimulating chondrocytes with interleukin IL-1β or lipopolysaccharides (LPS) increases protein levels of inflammatory-associated proteins, such as complement components, and also matrix metalloproteinases that degrade extracellular matrices. This increase is attenuated by chondroitin supplementation (Chan et al., 2005; Monfort et al., 2005; Tahiri et al., 2007; Legendre et al., 2008; Campo et al., 2009; Calamia et al., 2010; Imada et al., 2010; Calamia et al., 2012b), supporting a molecular role of chondroitin sulfate in blocking inflammation and extracellular matrix degradation (Figure 1). Similarly, in mice arthritis models, chondroitin sulfate slowed cartilage destruction and partially blocked inflammation (Omata et al., 2000). Mechanistically, chondroitin sulfate inhibits translocation of NF-ƙB, thereby decreasing NF-ƙB downstream signaling resulting in lower levels of pro-inflammatory cytokines and enzymes, such as IL-1β, IL-6, TNF-⍺, Cox-2, and Nos-2 (Jomphe et al., 2007; Campo et al., 2008; Xu et al., 2008; Souich et al., 2009; Vallières and Souich, 2010) (Figure 1).

Interestingly, across species, including humans, NF-ƙB increases in several tissues during aging (Tilstra et al., 2011). Genetic inhibitions of NF-ƙB delay several age-related pathologies in mice (Tilstra et al., 2011). Furthermore, in the hypothalamus of old mice, NF-ƙB activation decreases lifespan, whereas NF-ƙB inhibition increases lifespan (Zhang et al., 2013). Pharmacological inhibition of NF-ƙB also increases the lifespan of *Drosophila* (Moskalev and Shaposhnikov, 2011). This suggests that some beneficial effects of chondroitin sulfate supplementation might be mediated through inhibiting NF-ƙB age-related chronic inflammation resulting in improved health and longevity.

Taken together, supplementation of chondroitin sulfate increases lifespan *via* improving extracellular matrix homeostasis and *via* inhibiting chronic inflammation. Chronic inflammation can lead to upregulation of matrix metalloproteinase and extracellular matrix fragmentation and degradation as seen in osteoarthritis, suggesting a mechanistic link between disease progression and aging. However, chondroitin sulfate supplementation can increase the lifespan of model organisms *via* both mechanisms independently: *i.e.,* extracellular matrix remodeling or inhibiting chronic inflammation, suggesting distinct but also overlapping modes of action.

## Evidence of Chondroitin Sulfate Promoting Healthy Aging in Humans

### Uptake of Chondroitin Sulfate

Is orally supplemented chondroitin sulfate being absorbed by the body, and does it reach the target tissue? In rats and dogs, 70% of the orally administered radioactive-labeled chondroitin sulfate was found within 2 hours in the bloodstream and showed the highest concentrations in the intestine, liver, kidneys, synovial fluid, and cartilage after 24 h (Conte et al., 1995). In humans, about 20 μg/ml endogenously produced chondroitin sulfate is found in the blood circulation, and this level is constantly maintained without any effects of circadian rhythm (Jackson et al., 2009). Supplementing chondroitin sulfate with a single dose of 1,200 mg, as clinically used, did not reach a significant increase above endogenous chondroitin sulfate levels in the bloodstream within 2–4 h (Jackson et al., 2009), but a single dose of 4,000 mg of chondroitin sulfate doubled the chondroitin sulfate levels in the blood plasma within 2–4 h (Volpi, 2002). By contrast, other studies have reported repeated application of exogenous chondroitin sulfate peaks during 2–8 h upon intravenous, intramuscular, or oral routes (Conte et al., 1991a; Ronca and Conte, 1993; Conte et al., 1995; Volpi, 2003). Furthermore, daily doses of 800–1,200 mg of orally taken chondroitin sulfate significantly increased chondroitin plasma concentration within 24 h (Ronca and Conte, 1993; Conrozier, 1998). Of the orally taken chondroitin sulfate, about 30% of chondroitin sulfate (full-length and degraded) is excreted by the urine, whereas about 10% of the full-length and 20% of the degraded lower-molecular weight chondroitin sulfate is absorbed by the body (Conte et al., 1991b; Ronca and Conte, 1993; Ronca et al., 1998). Intact full-length chondroitin sulfate is taken up by cells via pinocytosis (McCarty et al., 2000). Orally taken chondroitin sulfate is absorbed in the proximal part of the small intestine (Souich, 2014). Probably most of the chondroitin sulfate is degraded in the colon and the cecum (Souich, 2014). After the partial excretion in the urine, chondroitin sulfate is mainly retained by the kidney and the liver (Pecly et al., 2006). However, accumulation of orally administered chondroitin sulfate in the joint tissue has been detected (Souich, 2014). Summing up, orally supplemented chondroitin sulfate is taken up by the body and reaches target tissues.

### Chondroitin Sulfate Impact on Age-Related Diseases

Endogenous chondroitin sulfate in the extracellular space affects growth factor and matrix metalloprotease reservoir storage, as well as their presentation and release. Chondroitin sulfate binds directly to cell surface receptors, such as L- and P-selectins and CD44 (Figure 1), and thereby modulates malignant transformation, metastasis, and tumor cell migration (Afratis et al., 2012). The use of chondroitin sulfate and glucosamine is also associated with a lower risk for various cancers, especially colorectal cancer, lung cancer, and adenocarcinomas (Satia et al., 2009; Kantor et al., 2016; Rani et al., 2018). Supplementation of chondroitin sulfate and glucosamine beneficially altered the gut microbiome (Navarro et al., 2019), suggesting a possible mode-of-action *via* improving microbiome composition to lower colorectal cancer incidences.

One of the most studied age-related diseases with regards to chondroitin sulfate supplementation is osteoarthritis, although it is still controversial how effective chondroitin sulfate is in slowing the disease progression. Osteoarthritis is a major health complication affecting >237 million middle-aged people worldwide (GBD 2015 Disease and Injury Incidence and Prevalence Collaborators, 2016). Osteoarthritis is the wear down of weight-bearing joint cartilage, such as knees, hips, and vertebrae. During aging, the self-repair of the extracellular matrix of joints declines resulting in insufficient repair upon mechanical stress and damage leading to cartilage degeneration, stiffness, pain, and chronic inflammation (Jerosch, 2011).

The consensus of many randomized clinical trials concluded that chondroitin sulfate supplementation showed a moderate effect on pain relief, larger efficacy on slowing the age-dependent shrinkage of the knee joint space, and improved knee function but also some considerable inconsistencies were observed across trials (Leeb et al., 2000; Jordan et al., 2003; Richy et al., 2003; Uebelhart et al., 2004; Michel et al., 2005; Clegg et al., 2006; Hathcock and Shao, 2007; Bruyère et al., 2008; Hochberg et al., 2008; Huskisson, 2008; Uebelhart, 2008; Kahan et al., 2009; Lee et al., 2009; Wildi et al., 2011; Zegels et al., 2013; Lomonte et al., 2018). A more detailed comparison is covered by these systematic reviews and meta-analyses (Wandel et al., 2010; Zhu et al., 2018; Honvo et al., 2019a; Fernández-Martín et al., 2021).

One contribution to the variable outcomes might be the manufacturing grade quality of chondroitin sulfate, which in some clinical trials, non-pharmaceutical grade chondroitin sulfate was included (Henrotin et al., 2014; Martel-Pelletier et al., 2015; Bruyère et al., 2017; Reginster and Veronese, 2021). Many tested food supplements had lower chondroitin sulfate concentration as indicated on their label and were less potent than pharmaceutical-grade chondroitin sulfate to inhibit inflammation markers *in vitro* (Stellavato et al., 2019). For instance, highly purified chondroitin sulfate administration reduced hand pain in a single-center, randomized, double-blind, placebo-controlled clinical trial (Gabay et al., 2011). Thus, it is imperative to use pharmaceutical-grade chondroitin sulfate for treatment (Martel-Pelletier et al., 2015; Honvo et al., 2019b; Reginster and Veronese, 2021). Another variable might be the time of application during the disease progression. For instance, for osteoarthritis, the sooner chondroitin sulfate was applied after diagnosis (i.e., earlier stages of the disease), the higher was the chance of success and beneficial response (Bruyère et al., 2020). Taken together, pharmaceutical-grade chondroitin sulfate might alleviate and slow age-related disease progression.

### Chondroitin Sulfate Use is Associated With Human Longevity

The question becomes whether chondroitin sulfate might be applicable as a preventative and geroprotective strategy in humans. There are three large cohort studies that showed a reduction in all-cause mortality of chondroitin sulfate users.

The first two studies examined the VITAL (Vitamins and Lifestyle) prospective cohort, which included both men and women aged 50–76. 77,718 people were examined for their use of vitamins, minerals, and other supplements in relation to mortality. These studies revealed that after 5 and 6.8 years of follow-up that the multivariate-adjusted hazard ratio of chondroitin sulfate users (>4 days/week for >3 years) were 0.83 and 0.86 compared to non-users, suggesting a 17 and 14% significant decrease in risk of total mortality, respectively (Pocobelli et al., 2010; Bell et al., 2012).

The third study examined 16,686 people of the US NHANES cohort. This study found that after an 8.9-years follow-up, the multivariate-adjusted hazard ratio of a combinatory use of chondroitin sulfate with glucosamine was 0.73 for all-cause mortality and 0.42 for cardiovascular mortality compared to non-users, suggesting a 27% and a 58% lower likelihood of overall and cardiovascular mortality (King and Xiang, 2020). Thus, these longitudinal studies link chondroitin sulfate supplementation to human longevity.

## Perspectives

Chondroitin sulfate supplementation is associated with reducing all-cause mortality in humans and increasing the lifespan of model organisms. But many gaps remain in our understanding of how chondroitin sulfate supplementation improves health during aging. Possible mechanisms include the reduction of chronic age-related inflammation and enhancement of extracellular matrix homeostasis. However, we need to define the missing steps in linking these two and possibly other mechanisms of chondroitin sulfate action to improve healthy aging. Such studies will provide new insights and help the development of therapeutic approaches that need to be tested in controlled clinical trials of chondroitin sulfate supplementation in age-matched individuals. Perhaps individualized assessment of chondroitin sulfate deficits might allow a personalized medical approach of chondroitin sulfate supplementation with other geroprotective drugs.
